# Supplementary material for: Solution-processable polymers of intrinsic microporosity for gas-phase carbon dioxide photoreduction
Source: Nat Commun. 2023 Jun 10;14:3443. doi: 10.1038/s41467-023-39161-6 (PMC10257718; doi:10.1038/s41467-023-39161-6)
Supplement: Supplementary file 1 — Supplementary Information [file 41467_2023_39161_MOESM1_ESM.pdf]

Supplementary Information on Solution-Processable Polymers of Intrinsic Microporosity for Gas-Phase Carbon Dioxide Photoreduction.

Floriana Moruzzi [1], Weimin Zhang [2], Balaji Purushothaman [2], Soranyel Gonzalez-Carrero [3], Catherine M. Aitchison [1], Benjamin Willner [1], Fabien Ceugniet [1], Yuan Bao Lin [1], Jan Kosco [2], Hu Chen [4], Junfu Tian [1], Maryam Alsufyani [1], Joshua S. Gibson [5], Ed Rattner [6], Yasmine Baghdadi [6], Salvador Eslava[6], Marios Neophytou [2], James R. Durrant [3], Ludmilla Steier [1], Iain McCulloch [1]

[1] Department of Chemistry, Oxford University, Chemistry Research Laboratory, 12 Mansfield Road, Oxford, OX1 3TA
[2] KAUST Solar Centre, King Abdullah University of Science and Technology (KAUST), Thuwal, 23955, Kingdom of Saudi Arabia.
[3] Department of Chemistry and Centre for Processable Electronics, Imperial College London, 80 Wood Lane, London, W12 7TA, UK.
[4] School of Physical Sciences, Great Bay University, Dongguan 523000, China.
[5] Henry Royce Institute Oxford Energy Storage Centre, Department of Materials, University of Oxford, Parks Road, Oxford, OX1 3PH, UK
[6] Department of Chemical Engineering, Imperial College London, LondonSW7 2AZ, UK

Supplementary Methods

Characterisation data collection and analysis: ^1H NMR spectra were recorded at ambient temperature on a 400 MHz Bruker spectrometer. Chemical shifts were reported in units of parts per million (ppm, δ) in solutions of $CDCl_3$, and referenced using the residual $CDCl_3$ (7.26ppm) as an internal standard. Where interpretable, coupling constants (*J*) are reported in Hz and splitting patterns reported as s (singlet), d (doublet), t (triplet) and m (multiplet). All NMR data were analysed using MestReNova software. Molecular Weight data was recorder on an Agilent $CHCl_3$ GPC (1200 series).UV-Vis absorption spectra were recorded on a Shimadzu UV-1800 spectrometer. PESA spectra were recorded on a KP Technology APS02 system over wavelengths of ca. 180 – 280 nm. For both thin film UV-Vis and for PESA, samples were prepared by drop-casting or spin-coating from a concentrated solution (ca. 5 mg mL^{-1}) of polymer in chloroform onto ITO-coated glass slides. BET sorption and pore size data were recorded on a Micromeritics 3Flex Surface Characterization Analyzer using multi-point surface area analysis. The pore size distribution was estimated by the desorption isotherm, using the Barrett, Joyner, and Halenda (BJH) method. Agilent 1200 GPC in chloroform was used to collect information about molecular weight of the polymers. TEM was carried out on a JEOL-2100 TEM . Surfaces were formed by spincoating films on ITO glass and subsequently studied by X-ray photoelectron spectroscopy (XPS) using a water-cooled monochromatic Al Kα source (hv = 1486.6 eV, 15 kV anode voltage, 25 W beam power). Charge compensation with the in-built e^- flood-gun was utilised to prevent peak broadening and position changes induced by charging (1.35 V electron energy, 20 µA beam current). Core level spectra were collected with a pass energy of 55 eV and a step size of 0.1 eV / step (giving a Ag 3d5/2 peak with fwhm of 0.69 eV). The analysis chamber pressure was maintained below 5×10^{-9} mbar during measurement. Data analysis was performed using the CasaXPS software package. The glass substrate was weighed out before and after deposition.). Polymer samples (between 2.5 mg and 5 mg) were digested in nitric acid (2 mL) and then analysed by ICP-MS. ICP-MS was run on a Perkin Elmer NEXION 2000B.

Photocatalysis measurements: The Photocatalytic Reactor Set-up was custom built; the light was provided by a 300-W Xe lamp (Asahi Max 303) fitted with an ultraviolet–visible mirror module (350–800 nm) and calibrated to 1 sun using an Ocean Optics USB2000 spectrometer. The amounts of gaseous photoreduction products were recorded by an Agilent 8860 GC system, equipped with TCD and FID detectors. Calibration was carried out using a custom-made reference gas containing 1000 ppm of CO_2, CO and CH_4 (BoCPolymers were drop cast on a glass slide, loaded into the reactor and vacuum was applied for 3 hours. The system was evacuated and refilled 6 times with CO_2 and H_2 gasses in the ratio 3:1 (H_2:CO_2). The pressure in the reactor was allowed to increase to 1.15 bar, after which the system was sealed and simulated sunlight at 1.5 A.M was irradiated on the polymer film. A rotary pump was employed as part of the gas-tight set-up to ensure flow of gasses through the system, and the mixture was injected every hour into a GC flowing in nitrogen, equipped with FID detector and Jetanizer. For stability studies, the chamber was evacuated and refilled with CO_2 and H_2 every 24 hours, the sample was left in the chamber. The illumination area and mass of polymers were kept as constant as possible to allow for close comparison between the polymers. Carbon nitride was commercially purchased from Nanoshel and tested as a standard.TiO_2 P25 nanoparticles were purchased from Sigma Aldritch. $^{13}CO_2$ Photocatalysis Measurements: Carried out on the Eslava Group set-up (Imperial College London), at atmospheric pressure, $^{13}CO_2$ (BOC, >99.9 % atom) was flushed through the reactor with hydrogen, the 35 mL reactor was closed and 1.5 AM sun was shone overnight. The evolved gasses were analysed by a mass spectrometer (Shimadzu MS) equipped with a Q-bond and a MolSieve column with gas sampling valves connected directly to the photoreactor. **Time-Correlated Single-Photon Counting (TCSPC):** The PL lifetime of polymers thin-films were measured using time-Correlated Single-Photon Counting (TCSPC) setup (Horiba DeltaFlex), equipped with a pulsed LED excitation source (404 nm, Horiba NanoLED series) and a fast rise-time photomultiplier detector (Horiba PPD-650). The instrument response function (IRF) was measured at the excitation wavelength. A suitable long-pass filter was inserted between the sample and detector to block off scattered excitation light in all the measurements. **Palladium scavenging** The polymers were dissolved in chloroform (100 mg in 100 mL) and added to an equal volume of aqueous solution of sodium diethyldithiocarbamate (1 g in 100 mL). The biphasic mixtures were stirred vigorously for 5 hours at 60 °C. The solutions were then allowed to cool to room temperature. The organic layers were collected and washed three times with deionised water. This entire process was repeated six times for each polymer. The final chloroform fractions were then filtered through a glass frit packed with Celite®. The resulting solutions were concentrated under reduced pressure, precipitated into methanol, and filtered using a PTFE filter. The polymers were then dried *in vacuo*.

AQY calculations [1]

The Apparent Quantum Yield was calculated using the equation below:

$$AQY\ (\%) = \frac{2 \times N_A \times h \times c}{P \times S \times \lambda_m \times t_s} \times 100 \qquad (1)$$

Where and N_A is Avogadro's number (6.022×10^{23} atoms mol^{-1}). h refers to Planck's constant (6.626×10^{-34} J·s), c is speed of light (3×10^{8} m s^{-1}). P is the power in Wm^{-2} , S the illuminated area, λ_m the wavelength and t_s the time in seconds.

Supplementary Experimental Details

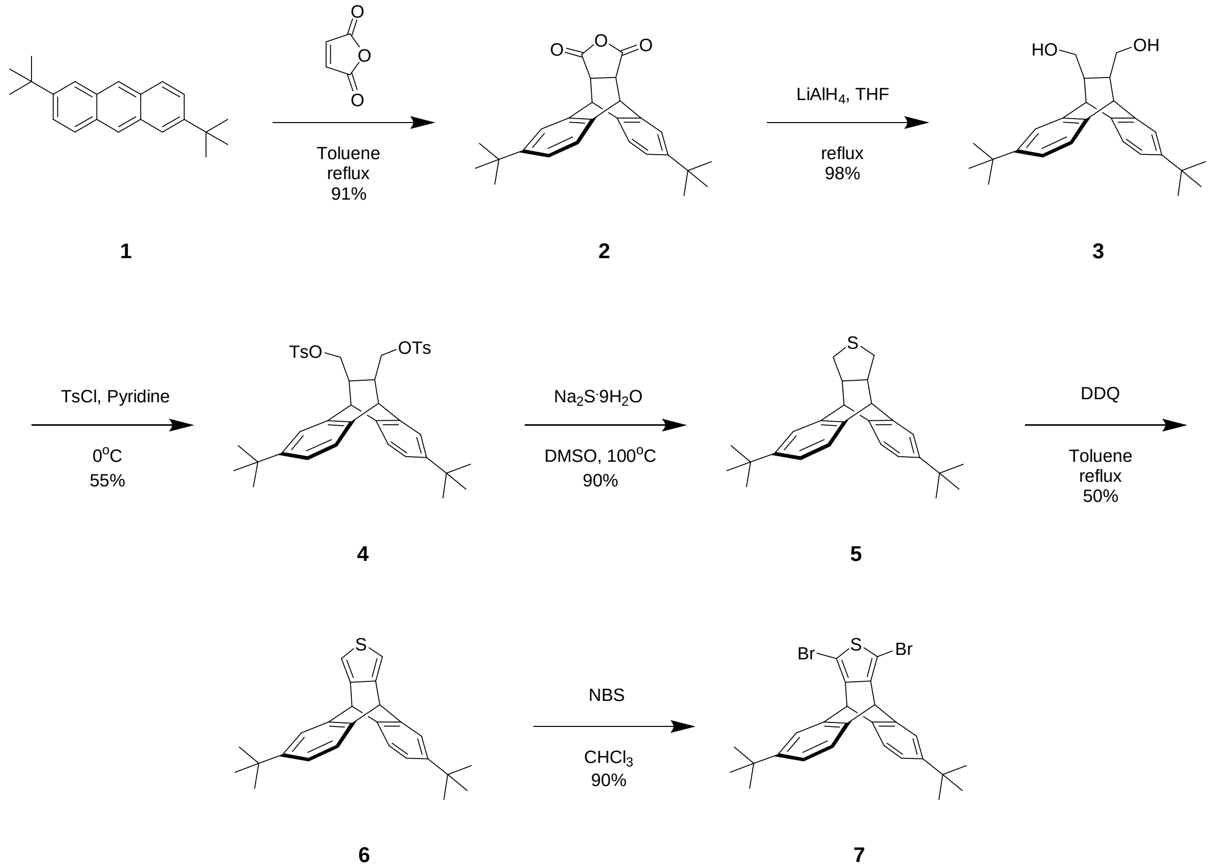

Fig S1: Synthesis of monomer 7. Scheme showing the steps in the synthesis of monomer 7

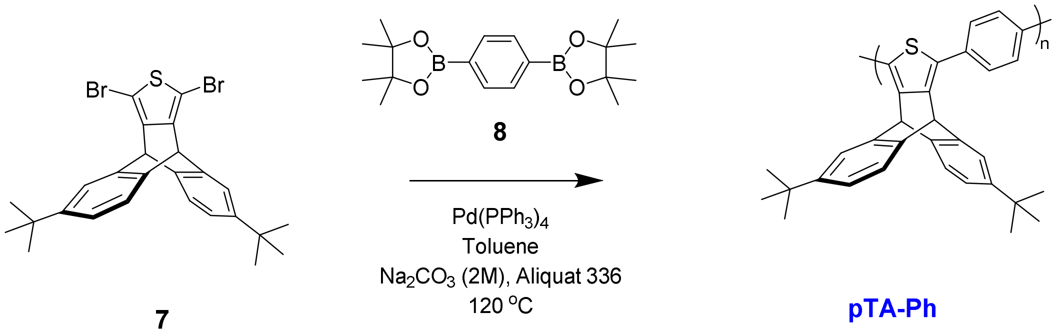

Fig S2: Polymerisation of pTA-Ph. Scheme showing the polymerisation step for pTA-Ph

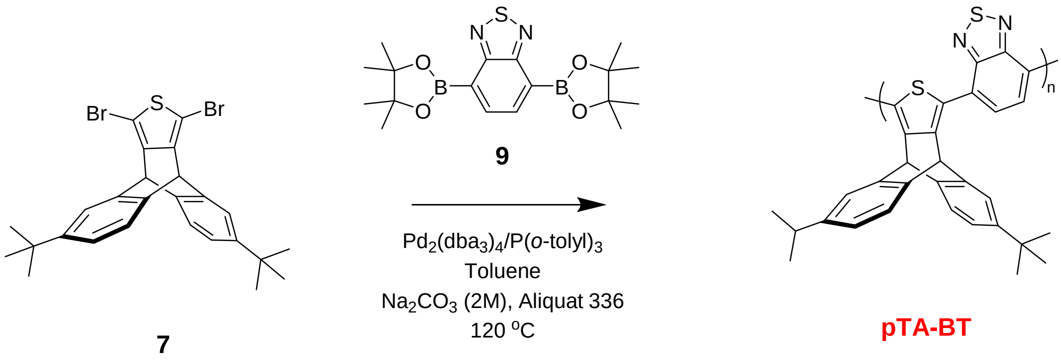

Fig S3: Polymerisation of pTA-BT. Scheme showing the polymerisation step for pTA-BT

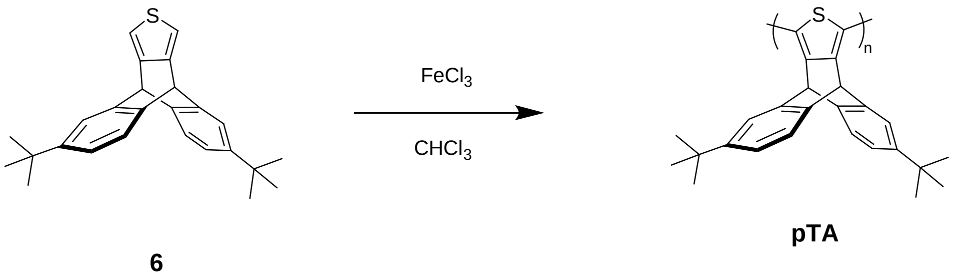

Fig S4: Polymerisation of pTA. Scheme showing the polymerisation step for pTA

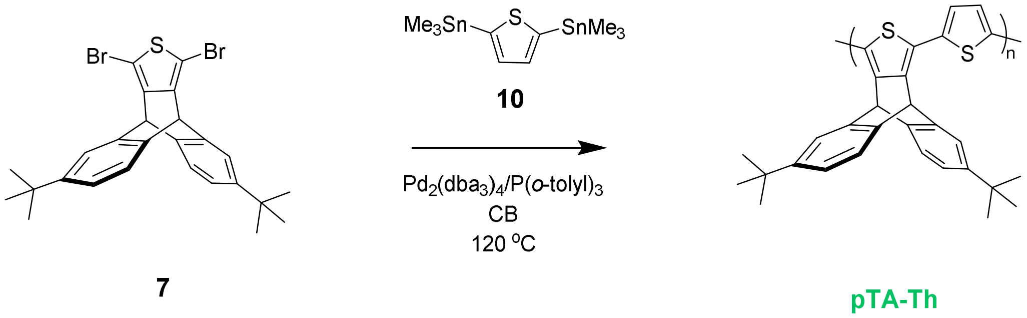

Fig S5: Polymerisation of pTA-Th. Scheme showing the polymerisation step for pTA-Th

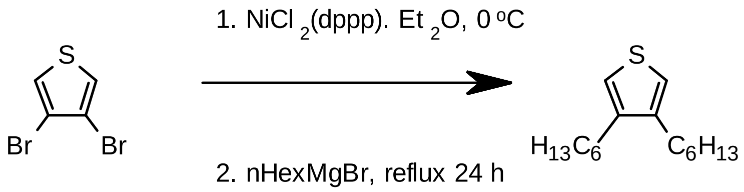

Fig S6: Synthesis of 3,4-dihexylthiophene. Scheme showing the synthesis of the monomer used to make pDHT, with experimental:

To a suspension of $NiCl_2$(dppp) (292 mg, 0.53 mmol) and 3,4-dibromothiophene (3 ml; 6.56 g, 27.1 mmol) in Et_2O (70 ml) at 0 °C, was added dropwise a concentrated solution of *n*HexMgBr in Et_2O, (67.5 ml, 135 mmol). At the end of the addition, the mixture was let to reach RT then heated to reflux for 24h. Afterwards, a 1M HCl aqueous solution (10 mL) was added carefully to the mixture under vigorous stirring. After 30 min, the resulting mixture was filtered over celite, and the filtrate evaporated to dryness. The residue was then extracted with DCM. washed with brine, water and dried over $MgSO_4$. The solvent was removed under vacuum. The crude was pre-purified by filtration over a small plug of silica gel (eluent: Petroleum spirit 100%). Purification was achieved by distillation under reduced pressure (150ºC, $9 \cdot 10^{-2}$ mbar) to afford the monomer (5.81 g) in 85% yield as a colourless oil.^{1}H NMR ($CDCl_3$, 400 MHz) δ(ppm): 6.89 (s, 2H), 2.56-2.47 (m, 4H), 1.71-1.56 (m, 4H), 1.43–1.26 (m, 12H), 0.91 (t, 6H). [2]

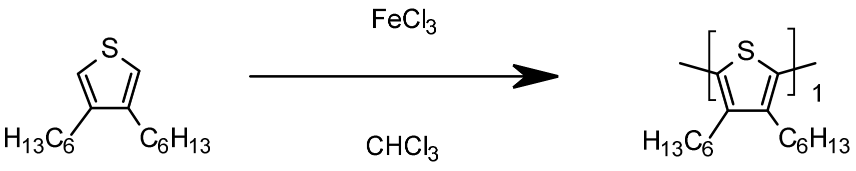

Fig S7: Polymerization of 3,4-dihexylthiophene with $FeCl_3$ Scheme showing the polymerisation step for pDHT, with experimental: [3]

A microwave vial was charged with Anhydrous $FeCl_3$ (0.35 g, 2.2 mmol) and 3,4-hexylthiophene (0.25 g, 1.00 mmol) was added via a syringe to the suspension. Anhydrous chloroform (1 mL) was injected. The resulting solution was purged with N_2 for 30 min and the reaction was heated to 130°C for 24 hours. The reaction mixture was poured into methanol

containing 5% concentration of HCl aq. The precipitate was collected by filtration and washed with methanol then extracted via Soxhlet's with methanol, acetone, hexane and finally chloroform and the solvent was evaporated to give a black product. Yield: 0.17 g (75%). M_n= 24KDa. ^{1}H NMR (400 MHz, Chloroform-d) δ 2.58 ppm (s), 1.59-1.1 ppm (d), 1.28 ppm (m), 0.89 ppm (t).

Supplementary Figures

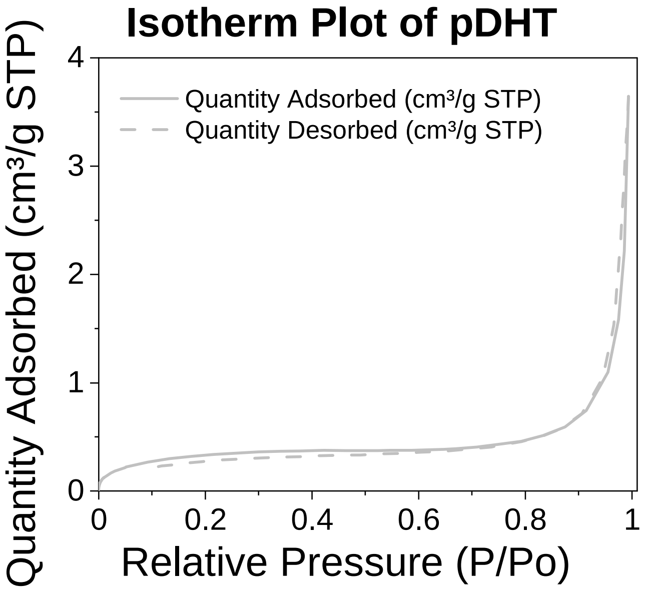

Fig S8. BET isotherm of pDHT. BET isotherm of pDHT, with BET surface area calculated as 29 m^2g^{-1}, this highlights the requirement for a robust orthogonal structure in order to create intrinsic porosity, since pDHT has amorphous alkyl chains rather than the iptycene core.

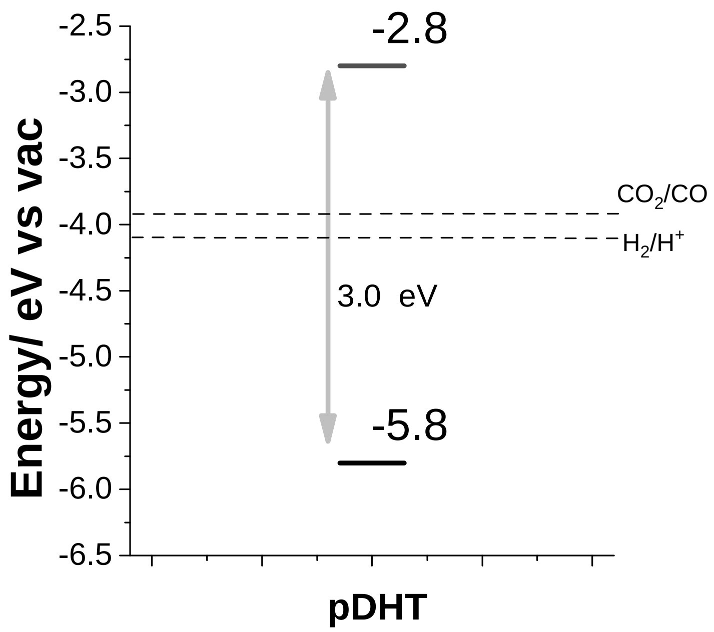

Fig S9. Energy levels of pDHT obtained by PESA and UV-Vis spectroscopy. Energy Level diagram obtained experimentally.

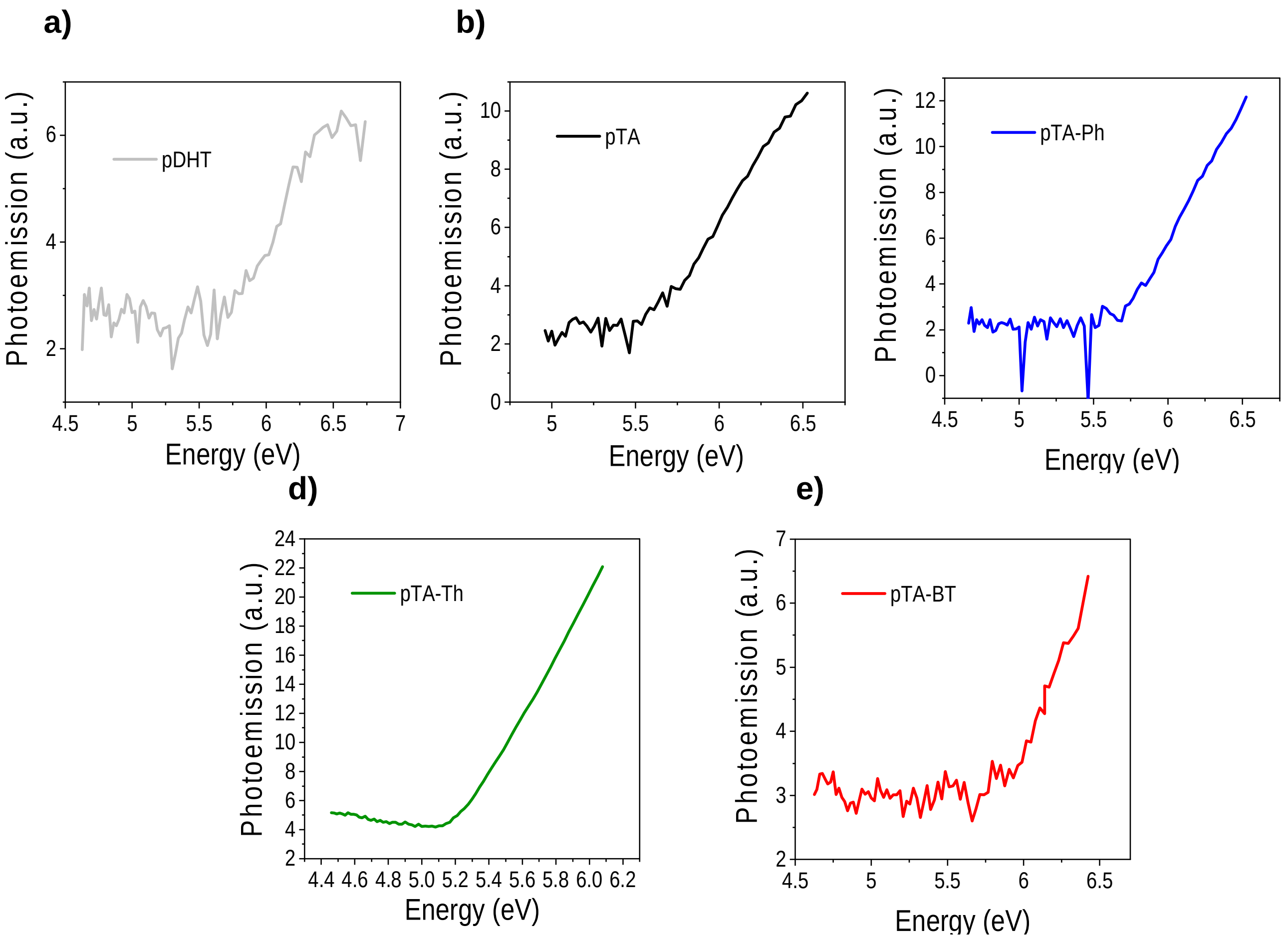

Fig S10. PESA of the polymers a) pDHT, b) pTA, c) pTA-Ph, d) pTA-Th, e) pTA-BT

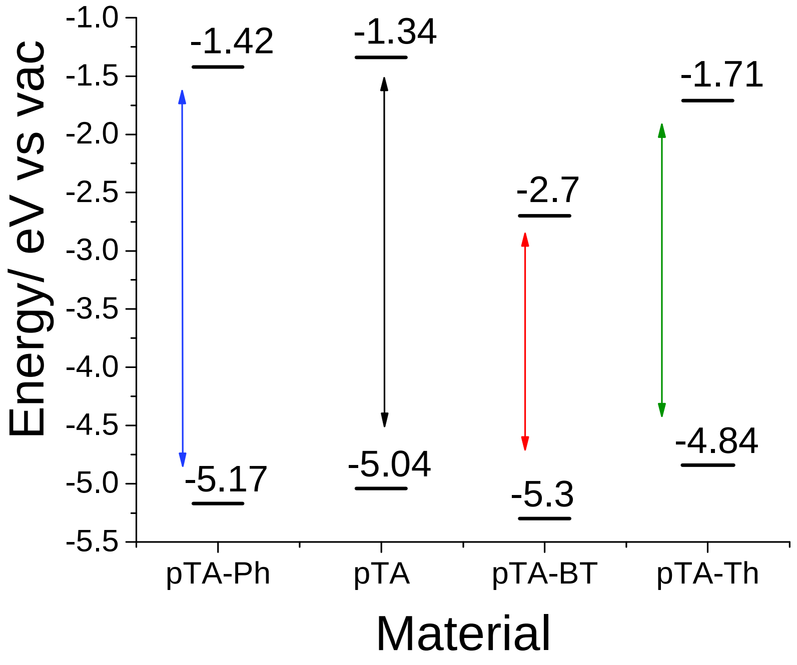

Fig S11. Diagram of predicted polymer energy levels by DFT To examine impact of chemical structure on the electronic delocalization and conformation of the backbone, the polymers were truncated as a trimer and simulated by density functional theory (DFT) simulations. The oligomers were first energy minimized with MMFF94 and further geometrically optimized with B3LYP-D3/6-31G(d,p) using Gaussian09.[4] Energy levels were in agreement in trend to experimentally obtained values, with only an increased red-shift of the pTA-Ph obtained experimentally.

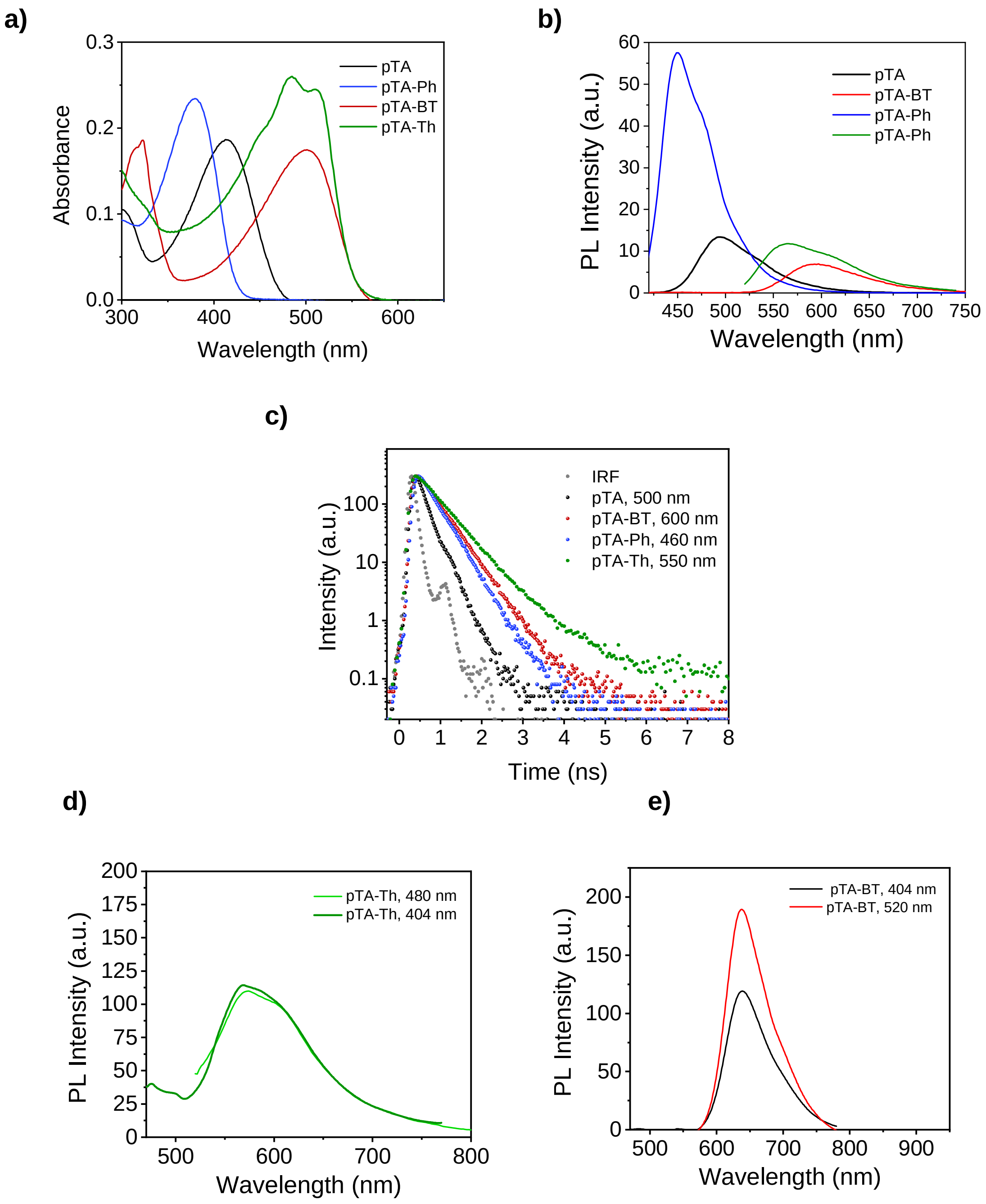

Fig S12. Supplementary UV-Vis and PL of polymers. a) UV-Vis of solutions of polymers in chloroform b) PL emission of polymers solutions in chloroform at 400 nm excitation wavelength, corrected by absorbed photons c) PL emission decay kinetics of polymers solutions upon 404 nm excitation wavelength and probed at the emission maximum d) PL emission of polymers films at different excitation wavelength for pTA-Th e) PL emission of polymers films at different excitation wavelength for pTA-BT

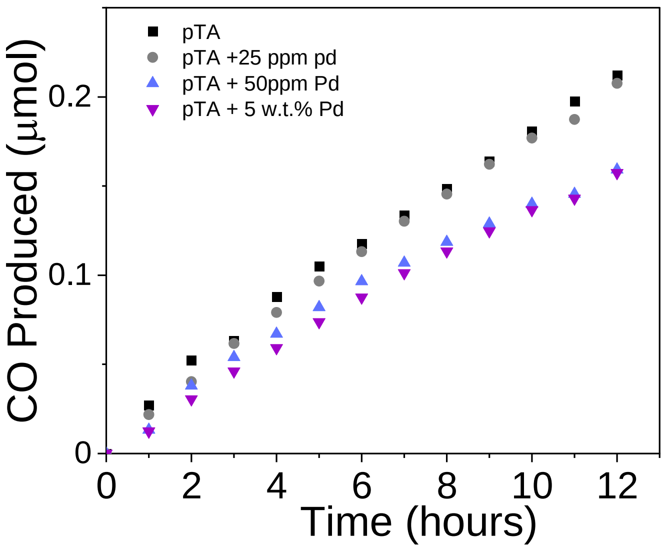

Fig S13. Kinetic curves of pTA with added amounts of palladium. Kinetic curves of pTA with different amounts of palladium added.

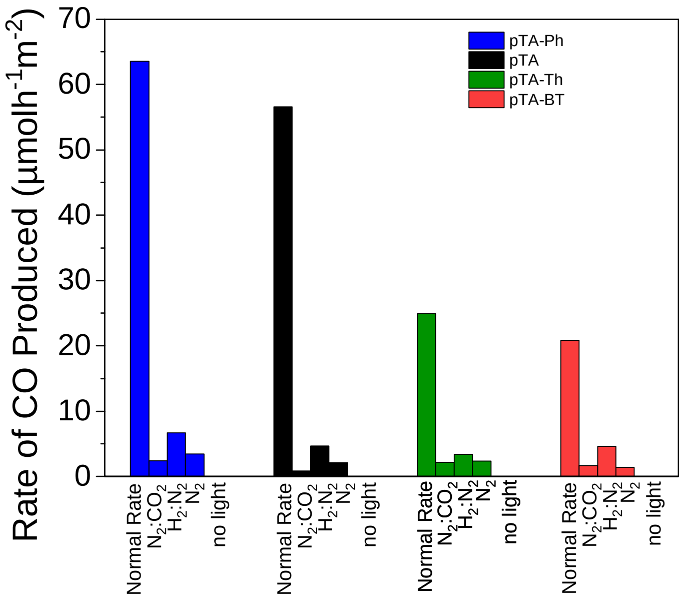

Fig S14. Rates of CO production showing control conditions. Firstly, rate obtained in testing conditions, then replacing H_2 with N_2, then replacing CO_2 with N_2, then in inert atmosphere and finally with normal conditions but under no illumination.

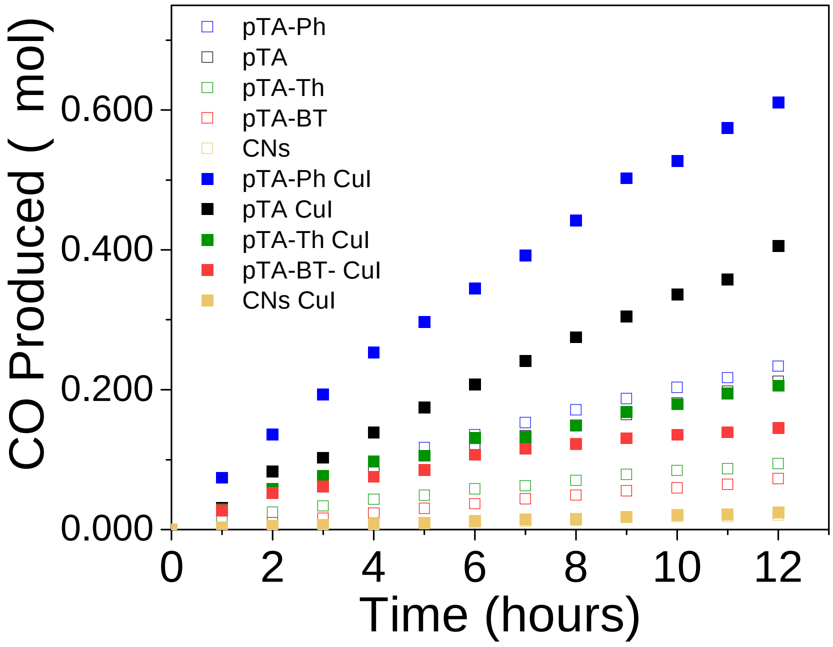

Fig S15. CO production over time from the polymers with no added co-catalyst compared with 5% CuI (with respect to Copper). CO_2 and H_2 were in a 1:3 ratio and at 1.15 bar.

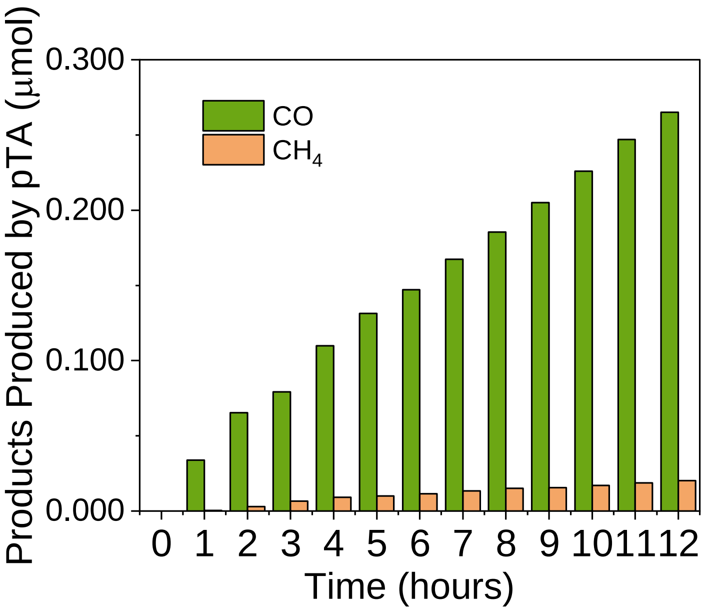

Fig S16. Rate of formation of CO and CH_4 by pTA. Graph showing product selectivity in pTA

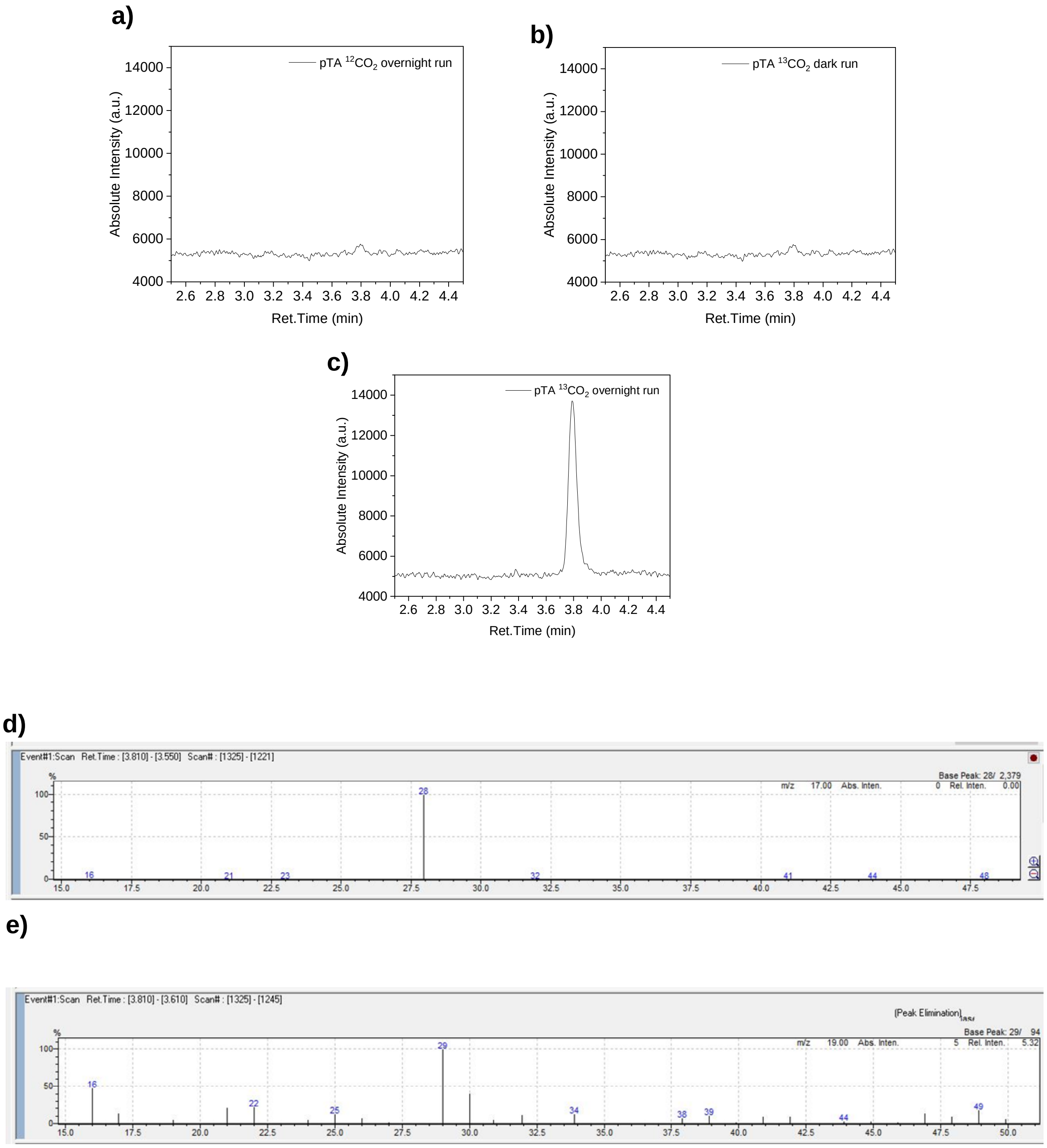

Fig S17. Figure showing $^{12}CO_2$ and $^{13}CO_2$ measurements. Mass spectrum chromatogram of pTA illustrating the ^{13}CO (m/z = 29) peak observed with a) $^{12}CO_2$ overnight, b) $^{13}CO_2$ at time zero and c) $^{13}CO_2$ overnight, showing the ^{13}CO (m/z = 29) peak observed. d) shows the mass spectrum of ^{12}CO e) shows the mass spectrum of ^{13}CO.

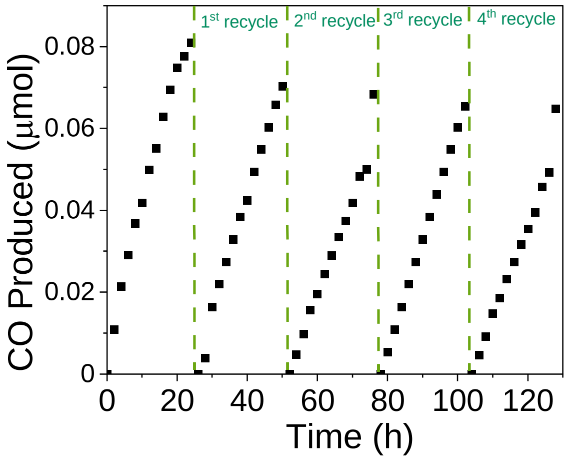

Fig S18. Stability Study for pTA-Ph. Study showing the activity of pTA-Ph, on a film containing 0.1 mg of polymer, over a 120 hour period.

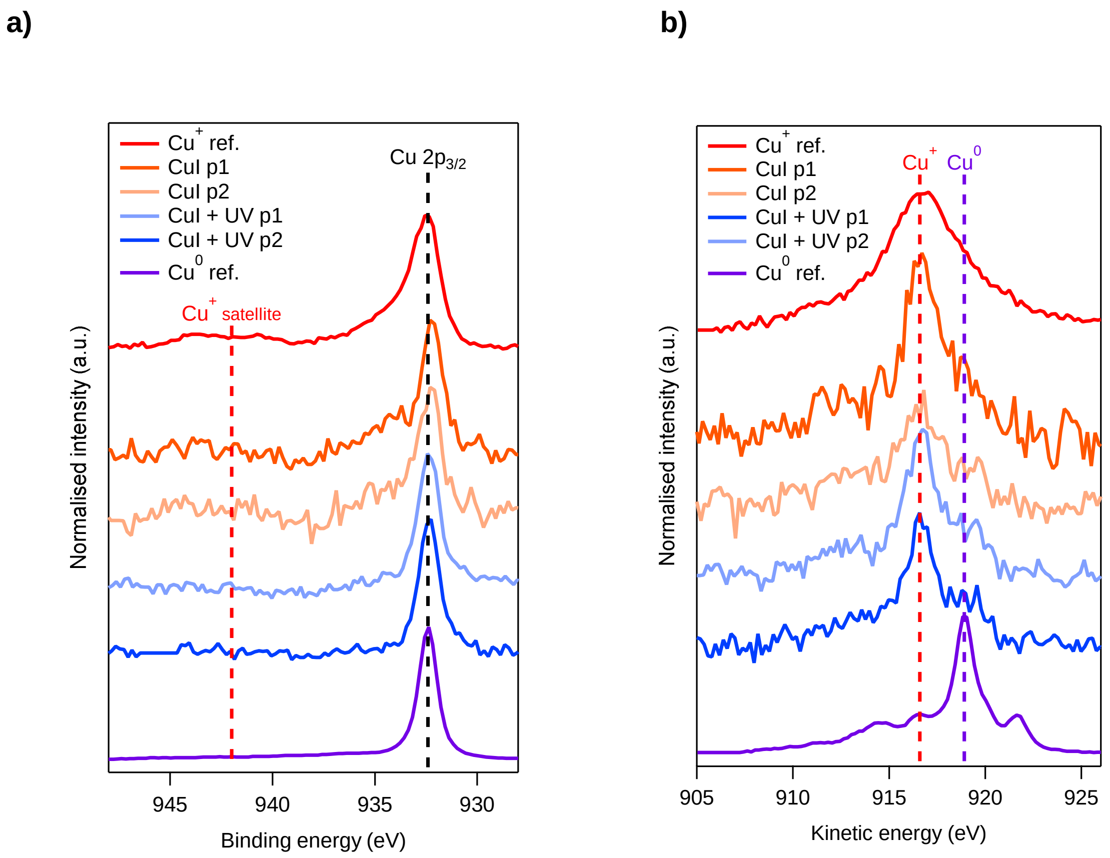

Fig S19. XPS data for copper studies. a) XPS of copper iodide b) reprised from Figure 6a Photoelectron spectra for the Cu $2p_{3/2}$ region, on two CuI samples pre-exposure (orange) and following UV+H_2 exposure (blue). All spectra display the principal peak at 932.4 eV, for the energy referencing used in this study, with the pre-exposure CuI samples showing a weak satellite feature (939 to 936 eV) attributed to Cu(I). Reference spectra are shown for Cu_2O (red) and pristine copper metal prepared by argon ion etching under ultra-high vacuum (purple).XPS measurements of copper iodide samples pre- and post-exposure demonstrate differences in the spectral features of the Cu LMM Auger (Figure 6a) and the Cu 2p photoelectron (Figure S12) transitions. The Cu LMM Auger peaks of two different CuI samples pre-exposure (orange) show a dominant peak at 916.6 eV, owing to the presence of Cu(I), with peak position consistent with a Cu_2O reference material (red). Following UV+H_2 exposure, CuI samples (blue) exhibit a 20% increase of a secondary peak at 919.0 eV, relative to the dominant peak at 916.6 eV. This change is consistent with an increase in the presence of Cu(0), as shown by the reference spectrum of argon ion etched copper metal (purple). The Cu $2p_{3/2}$ photoelectron peaks, at 932.4 eV (Figure S12), show a weak satellite feature (939 to 936 eV) attributed to Cu(I) which is suppressed following UV+H_2 exposure, however this cannot be ruled out as the presence of low quantities of Cu(II). [61] Peak positions and spectral shapes are consistent with XPS literature, for the binding energy referencing used in this work. The increase in component at 919.0 eV in the Cu LMM Auger peak is attributed to the conversion to Cu metal, following UV+H_2 exposure of the pure CuI samples. The same peak is not observed in the samples which were not exposed to operating conditions, supporting the notion that copper metal is being formed during the reaction.[5,6]

a) **b)**

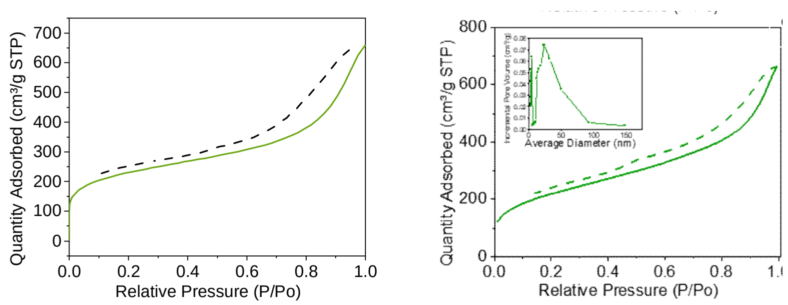

Fig. S20. BETs of pTA-Th taken 2 years apart, a) shows the curve obtained for pTA-Th in February 2020, b) shows the curve obtained from pTA-Th in March 2022, the surface area obtained was within an error of 10%.

Supplementary Tables

Table S1. DFT predicted HOMO and LUMO distributions of polymers. DFT predicted HOMO and LUMO distributions of polymers

Polymer	HOMO	LUMO
**pTA-BT**	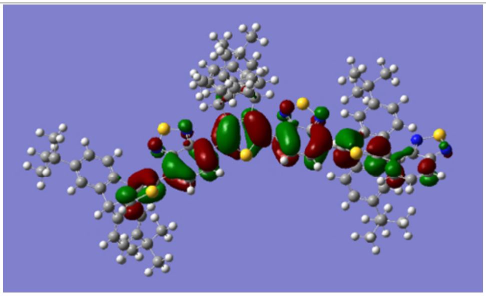	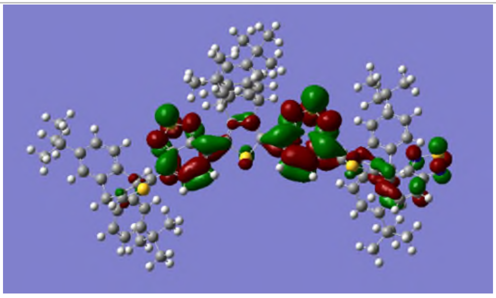
**pTA-Ph**	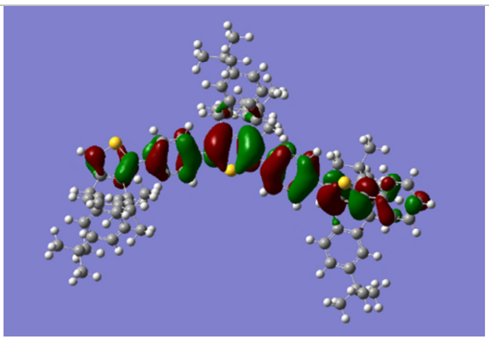	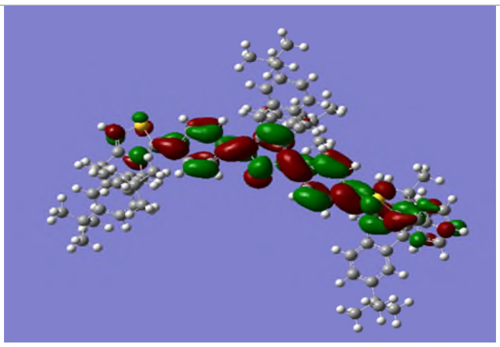
**pTA-Th**	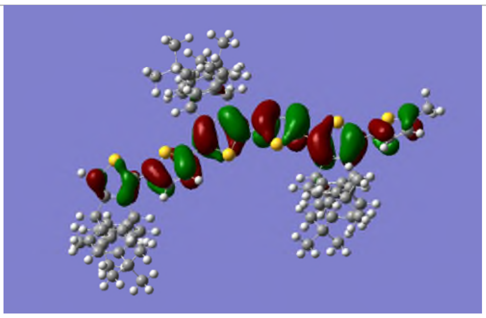	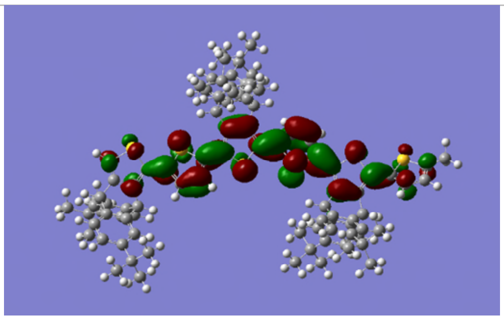
**pTA**	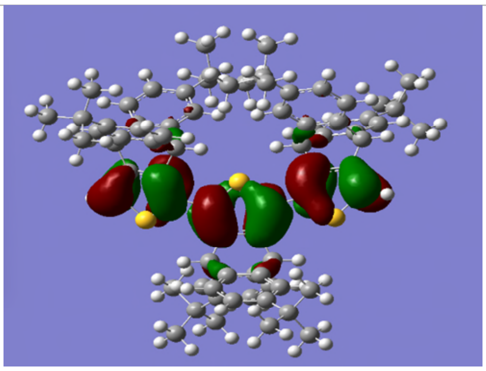	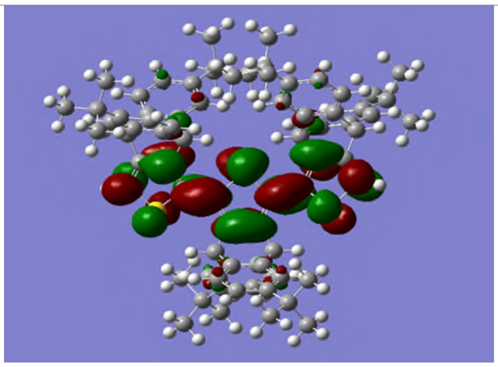

Table S2. Table showing calculated lifetimes of polymers. At their emission maximum, the PL emission decays were adjusted at bi or tri-exponential function. The average lifetime t_{avg} was calculated as according to $t_{avg} = \sum_i B_i t_i^2 / \sum_i B_i t_i$.

Polymer	λ emission (nm)	t_1 (ns)	t_2 (ns)	t_3 (ns)	B_1 (%)	B_2 (%)	B_3 (%)	X^2	t_{avg}[a]
**pTA**	550	0.12	0.34		88.3	11.7		1.00	**0.18**
	600	0.21	0.64	2.50	81.5	16.2	2.3	1.02	**0.75**
**pTA-Ph**	460	0.24	0.46	3.39	76.5	23.0	0.5	0.99	**0.50**
	550	0.30	1.02	4.14	73.8	17.8	8.4	1.19	**2.34**
**pTA -Th**	550	0.24	0.55		26.8	73.2		1.04	**0.51**
	640	0.28	0.04	0.88	46.4	38.1	15.5	1.04	**0.56**
**pTA BT**	640	0.27	0.54		65.6	34.4		1.04	**0.41**

Table S3. Supplementary Table of rates. Rates of reaction are with different units for comparison.

Polymer	Rate (μmol h^{-1})	Rate (μmol $h^{-1}g^{-1}$)	Rate (μmol $h^{-1}m^{-2}$)	Rate with CuI (μmol h^{-1})	Rate with CuI (μmol $h^{-1}g^{-1}$)	Rate with CuI (μmol $h^{-1}m^{-2}$)
**pTA**	0.017 ±0.001	8.5 ± 0.5	53 ± 3	0.033 ±0.003	17 ± 1.5	106 ± 10
**pTA -Ph**	0.019 ±0.001	9.5 ± 0.5	66 ± 4	0.05 ±0.01	25 ± 5	175 ± 35
**pTA -Th**	0.007 ±0.001	3.7 ± 0.5	25 ± 3	0.016 ±0.005	8 ± 2.5	55 ± 17
**pTA -BT**	0.006 ±0.001	3.1 ± 0.5	21 ± 4	0.015 ±0.002	6 ± 1	37 ± 6
**pDHT**	0.002 ±0.0006	0.9 ± 0.3	10 ± 3	-	-	-
**CNs**	0.002 ±0.0004	0.9 ± 0.2	6 ± 2	0.88 ± 0.2	1 ± 0.3	7 ± 2
**TiO_2**	0.005 ±0.001	2.1 ± 0.5	21 ± 5	-	-	-

Table S4. Table of controls done on polymer performance. Showing rates at different control conditions.

Polymer	N_2:CO_2/ μmolh^{-1}m^{-2}	H_2: N_2/ μmolh^{-1}m^{-2}	N_2/ μmolh^{-1}m^{-2}	No light/ μmolh^{-1}m^{-2}
**pTA**	2.4	6.7	3.5	-
**pTA-BT**	0.9	4.5	2.1	-
**pTA-Ph**	2.2	3.4	2.3	-
**pTA-Th**	1.67	4.6	1.4	-

Table S5. Amount of palladium and iron in the polymers following scavenging. Calculated by ICP-MS.

Polymer	Pd (ppm)	Fe (ppm)
**pTA**	-	26
**pTA -Ph**	15	-
**pTA -Th**	23	-
**pTA -BT**	31	-

Table S6. Calculated rates of pTA with added amounts of palladium. Showing rate effect at different palladium loadings

Polymer	Rate (μmol $h^{-1}g^{-1}$)
**pTA**	8.49
**pTA + 25 ppm Pd**	8.48
**pTA + 50 ppm Pd**	6.57
**pTA + 5 w.t.% Pd**	6.52

Table S7: XYZ Coordinates of Optimized Trimer Structure. For pTA, pTA-Ph, pTA-Th and pTA-BT.

PTA

C	-0.93893100	3.97019500	-3.14605100
C	0.17330800	3.88264800	-2.37754700
C	0.07575800	2.88551200	-1.35574400
C	-1.10102600	2.19159200	-1.37305600
S	-2.12426700	2.80314600	-2.65956500
C	2.11313400	5.88012500	1.61206500
C	2.21394100	6.85543100	0.62156300
C	2.01961500	6.45781900	-0.71312800
C	1.74318400	5.14188700	-1.02587900
C	1.64335400	4.17621700	-0.01632800
C	1.82705600	4.54702700	1.30112600
C	1.34801800	2.77441800	-0.53076600
C	1.52839900	4.56956300	-2.42219500
C	2.42449900	2.45919300	-1.56342100
C	2.53442800	3.42597800	-2.56662400
C	3.47645600	3.25642700	-3.56752200
C	4.28895700	2.12230400	-3.56596700
C	4.17084300	1.13050900	-2.58688900
C	3.21414900	1.32254900	-1.58353200
C	5.06894600	-0.11320600	-2.63310600
C	2.51790300	8.32742100	0.92886800
C	4.86380900	-0.84145300	-3.97448900
C	6.54039100	0.32151300	-2.49881600
C	4.75876200	-1.10143400	-1.50021900
C	2.72965100	8.57811800	2.42767400
C	3.79773500	8.75323100	0.18450700
C	1.33651300	9.19638300	0.45692500
C	-0.85492900	-2.35419300	1.36945800
C	0.39820400	-2.89911100	1.35046700
C	0.59920900	-3.92327100	2.32934800
C	-0.50778200	-4.18132400	3.06586700
S	-1.81891300	-3.14687300	2.60228200
C	4.41518400	-1.71207400	3.72290700

C	4.18184800	-0.69201400	2.79499900
C	3.28534000	-0.95797600	1.75366000
C	2.66330200	-2.18946800	1.64967200
C	2.87614300	-3.18379100	2.60836400
C	3.76343100	-2.94344900	3.64392300
C	2.02964100	-4.43777900	2.38011300
C	1.66545000	-2.59122300	0.56940300
C	2.34887900	-4.90857200	0.96581400
C	2.14988600	-3.91768100	0.00280000
C	2.40590700	-4.19654900	-1.32911300
C	2.86383100	-5.46206700	-1.68744300
C	3.06957600	-6.46857000	-0.73803900
C	2.80050200	-6.16787400	0.60345900
C	3.57985000	-7.84313600	-1.19171600
C	4.90579700	0.65523100	2.92163200
C	4.96046600	-7.67739500	-1.85447900
C	3.72488100	-8.82674900	-0.02236300
C	2.59033500	-8.44757300	-2.20586800
C	4.67565200	1.24067400	4.32736900
C	6.41308600	0.43386700	2.69487700
C	4.40734500	1.68262100	1.89464800
C	-1.48102100	1.01499800	-0.60193000
C	-2.69971100	0.46680900	-0.32628100
C	-2.63265100	-0.79477700	0.34722100
C	-1.36175400	-1.20840800	0.62485600
S	-0.22477800	-0.02889700	0.01621300
C	-5.79219100	-2.43262100	-2.65809800
C	-5.86923600	-1.20129800	-3.30647600
C	-5.32999600	-0.08040200	-2.65104800
C	-4.74988700	-0.20335400	-1.40493800
C	-4.67921300	-1.44967300	-0.76852000
C	-5.20043800	-2.56281100	-1.39709900
C	-4.00273100	-1.40259900	0.59804000
C	-4.12446100	0.92246800	-0.59001300
C	-4.75151200	-0.35058600	1.40838500
C	-4.81162500	0.89583500	0.77144800

C	-5.44996800	1.94813300	1.39647800
C	-6.02879800	1.75782600	2.65543800
C	-5.97788500	0.52553500	3.30460500
C	-5.32226000	-0.53320700	2.65186500
C	-6.60877000	0.28338000	4.68177300
C	-6.50782100	-1.02431300	-4.68992900
C	-7.25246100	1.55158400	5.25765300
C	-7.69966900	-0.79686600	4.55283200
C	-5.52494000	-0.19508400	5.66643000
C	-7.06746000	-2.34049400	-5.24599000
C	-7.66677600	-0.01420200	-4.59065700
C	-5.44792300	-0.49530300	-5.67493400
H	-1.14122700	4.63528900	-3.97368400
H	2.25723400	6.14317600	2.65345100
H	2.08705200	7.18815800	-1.51529800
H	1.75050700	3.80876300	2.09443500
H	1.30356100	2.04250800	0.27433300
H	1.61371700	5.31860200	-3.21161900
H	3.58084700	3.99836700	-4.35433100
H	5.02440600	2.01219600	-4.35763900
H	3.06947900	0.57637400	-0.81227700
H	5.49040900	-1.73878900	-4.01888600
H	3.81931300	-1.14605700	-4.09361200
H	5.12699700	-0.20755900	-4.82604600
H	7.20120000	-0.55140300	-2.53519000
H	6.70462900	0.83524500	-1.54613900
H	6.83713500	1.00187500	-3.30229100
H	4.93800300	-0.66609600	-0.51241500
H	3.72200200	-1.45165200	-1.53381500
H	5.40044800	-1.98293000	-1.59011700
H	2.94308500	9.63824200	2.59584300
H	1.83828300	8.32451600	3.01023000
H	3.57474100	8.00301500	2.81910200
H	4.02581200	9.80421500	0.39267500
H	3.69273100	8.64412600	-0.89855800
H	4.65157800	8.14660600	0.50157300

H	0.41556400	8.91397100	0.97608200
H	1.53555000	10.25417700	0.66037000
H	1.16116200	9.08948500	-0.61741700
H	-0.64151100	-4.90251600	3.85976800
H	5.11450800	-1.54892100	4.53766900
H	3.05502600	-0.19776000	1.01774300
H	3.94863600	-3.70437800	4.39700700
H	2.19080400	-5.20753100	3.13716700
H	1.54981100	-1.83469700	-0.20492100
H	2.25104700	-3.43596400	-2.08945000
H	3.06211500	-5.66246000	-2.73610100
H	2.94295600	-6.91527000	1.37663300
H	5.34074400	-8.64764500	-2.19240200
H	4.91516900	-7.01476900	-2.72339300
H	5.68097200	-7.25441700	-1.14752300
H	4.08796400	-9.78907600	-0.39643700
H	2.76750300	-9.00471400	0.47774400
H	4.44412200	-8.46962700	0.72165700
H	2.48017400	-7.81625900	-3.09188600
H	2.94072900	-9.43074500	-2.53861400
H	1.60047900	-8.57013900	-1.75567300
H	5.18799600	2.20362600	4.42488500
H	3.60853200	1.40036100	4.51029900
H	5.05570400	0.58156800	5.11277500
H	6.95471700	1.38254100	2.77567300
H	6.59715800	0.01662100	1.69954300
H	6.83101800	-0.25911600	3.43133900
H	3.32984400	1.85514800	1.98623700
H	4.90717300	2.64227000	2.05677500
H	4.61769800	1.37901900	0.86467600
H	-6.19549200	-3.32214300	-3.12769500
H	-5.36370500	0.89870400	-3.12147100
H	-5.15072700	-3.53684300	-0.91873100
H	-3.97416000	-2.37796100	1.08417300
H	-4.19677100	1.89558100	-1.07633800
H	-5.50288100	2.92127500	0.91663100

H	-6.52257900	2.60093100	3.12423500
H	-5.25474100	-1.51011400	3.12326300
H	-7.68378900	1.33260900	6.23931800
H	-6.51808900	2.35272200	5.38869400
H	-8.05955000	1.92245800	4.61792800
H	-8.16903900	-0.98274100	5.52501800
H	-7.28880200	-1.74416400	4.19197700
H	-8.47680400	-0.47964000	3.85075100
H	-5.05611100	-1.12590700	5.33527700
H	-4.73728900	0.55734300	5.77124000
H	-5.96328700	-0.37396900	6.65423500
H	-7.51700800	-2.16325400	-6.22795900
H	-6.28339900	-3.09388000	-5.37186900
H	-7.84446400	-2.75500400	-4.59573600
H	-8.14063100	0.11859800	-5.56934900
H	-7.32210300	0.96688100	-4.25179000
H	-8.42668400	-0.36429200	-3.88533800
H	-4.61948000	-1.20412200	-5.76859800
H	-5.89004500	-0.34948300	-6.66660000
H	-5.03271900	0.46229100	-5.34802400

PTA-BT

C	-9.96132500	1.15440600	-3.35885600
C	-9.91009400	1.64935200	-2.09842400
C	-8.65063400	1.43484900	-1.45380200
C	-7.74936100	0.77671800	-2.23579500
S	-8.46229700	0.40460200	-3.79140900
C	-6.36499500	0.37943100	-1.95854800
C	-10.88313800	0.20612000	2.42793500
C	-12.12770800	0.39035300	1.81658200
C	-12.16342400	1.10870200	0.61463400
C	-10.99887900	1.61524300	0.06047700
C	-9.76465000	1.41811100	0.68513500
C	-9.70893000	0.70969100	1.87300900
C	-8.59151400	2.04175100	-0.06310600
C	-10.90021200	2.41158300	-1.23816200
C	-8.95010700	3.51318200	-0.25625700

C	-10.18477100	3.71070900	-0.88080900
C	-10.63193500	4.99902700	-1.11743000
C	-9.84505700	6.08077700	-0.72693700
C	-8.60824900	5.90486700	-0.09855000
C	-8.17244400	4.59294800	0.12992800
C	-7.78073400	7.13256900	0.30600500
C	-13.38997200	-0.18806600	2.47054500
C	-8.60746400	8.01867200	1.25683200
C	-6.47959200	6.74811800	1.02376500
C	-7.41537100	7.93597200	-0.95677700
C	-13.24226700	-1.71530300	2.60580000
C	-13.57314400	0.43678800	3.86653700
C	-14.65423800	0.09878900	1.64879300
C	-5.35226700	0.55479900	-2.86735200
C	-4.01439800	0.12701600	-2.63289600
C	-4.64099700	-0.68720600	-0.48464700
C	-5.99954300	-0.26234500	-0.72654800
N	-4.47098800	-1.24886400	0.71217600
S	-5.92459900	-1.24593200	1.43890400
N	-6.82235000	-0.53440100	0.28636700
C	-2.23698500	-0.92345000	-1.24209000
C	-1.75960800	-2.10566300	-0.76065600
C	-0.33376300	-2.17041600	-0.71195900
C	0.28010300	-1.03271800	-1.14211300
S	-0.91180300	0.14851100	-1.62564300
C	-3.61947100	-0.49491000	-1.47562600
C	1.70835600	-0.71009600	-1.20972400
C	-0.52144500	-6.26992400	-2.87701200
C	-1.91834500	-6.22109800	-2.92671400
C	-2.56846200	-5.29319400	-2.10280300
C	-1.84088100	-4.45859600	-1.27038200
C	-0.44579100	-4.52232500	-1.23415600
C	0.21544700	-5.43218300	-2.04150000
C	0.18638600	-3.52578700	-0.26769900
C	-2.42600500	-3.40234100	-0.33809400
C	-0.46877200	-3.77203300	1.08601100

C	-1.86695500	-3.69783100	1.04924100
C	-2.58684000	-3.88002600	2.21357600
C	-1.91384000	-4.13932600	3.41107200
C	-0.52268700	-4.22267100	3.46436000
C	0.19069200	-4.03471300	2.26957600
C	0.24653500	-4.52194000	4.75728200
C	-2.68121300	-7.15972400	-3.87137500
C	-0.67655200	-4.58554900	5.98144700
C	0.95909900	-5.87997900	4.61311100
C	1.29320900	-3.41894400	5.00617400
C	-2.27174800	-6.85738500	-5.32545400
C	-2.33624400	-8.62244100	-3.53350900
C	-4.20228800	-6.98911900	-3.75663600
C	2.29004300	-0.11499500	-2.30006200
C	3.67246500	0.22533500	-2.35247000
C	3.97781100	-0.63379300	-0.15155100
C	2.57680100	-0.97313500	-0.09621900
N	4.64144900	-0.96059900	0.95723200
S	3.55657700	-1.61704100	1.97315400
N	2.21492900	-1.52697800	1.06092500
C	5.97130100	0.30215600	-1.38979900
C	6.78001000	0.90660600	-0.47499000
C	8.14235700	1.01106300	-0.89263100
C	8.37307200	0.49667800	-2.13354700
S	6.89404400	-0.14944600	-2.80123300
C	4.54098600	-0.01129100	-1.31721000
C	9.61337700	0.43464500	-2.91486300
C	9.46530600	-0.29026500	3.36306000
C	8.13919100	-0.39194500	3.78223800
C	7.15266700	0.21490400	2.98816600
C	7.49021700	0.88231600	1.82833800
C	8.82738100	0.97392800	1.42154800
C	9.81288400	0.38741800	2.19059500
C	9.01620400	1.73717400	0.11457800
C	6.51756900	1.55933000	0.86952000
C	8.34228800	3.09143700	0.31306400

C	7.00709500	2.99486800	0.71239300
C	6.27376400	4.15025300	0.92271600
C	6.87983700	5.39137900	0.73737400
C	8.21665200	5.50845500	0.34278600
C	8.94004000	4.32769900	0.13040700
C	8.83589900	6.89974500	0.15362000
C	7.72151100	-1.12913700	5.06072400
C	8.07583700	7.64868400	-0.95730500
C	8.73263100	7.69195400	1.47062200
C	10.31667800	6.82822900	-0.24422800
C	8.91035000	-1.79922200	5.76251000
C	7.08586700	-0.12318800	6.03902000
C	6.69376200	-2.22319400	4.71304200
C	9.65625500	0.75089700	-4.24956500
C	10.85202700	0.67156700	-5.02809500
C	12.04005300	0.27561400	-4.49068300
C	12.05422800	-0.07481000	-3.11035900
C	10.84849600	-0.00228000	-2.32228400
N	13.11340400	-0.50499400	-2.42137000
S	12.58951700	-0.79403700	-0.90862500
N	11.02231800	-0.38893100	-1.05821200
H	-10.78106600	1.17748900	-4.06296500
H	-10.81947200	-0.34415500	3.36224700
H	-13.10275000	1.27947700	0.09983200
H	-8.75472900	0.54524500	2.36400900
H	-7.63802700	1.89616000	0.44194900
H	-11.86727000	2.57177700	-1.71846400
H	-11.58890500	5.16788500	-1.60326800
H	-10.21224200	7.08427000	-0.92032900
H	-7.22003500	4.40337200	0.61299200
H	-8.02529500	8.89551700	1.56068000
H	-9.52566500	8.37761200	0.78368400
H	-8.88841300	7.46491200	2.15794500
H	-5.92964000	7.65467200	1.29474700
H	-5.82632400	6.14425100	0.38600500
H	-6.67463600	6.19138600	1.94598100

H	-6.82369000	7.32575600	-1.64603000
H	-8.30752000	8.27627700	-1.49028700
H	-6.82700700	8.82054500	-0.68912900
H	-14.13864800	-2.14527600	3.06582900
H	-13.10331600	-2.18017600	1.62490300
H	-12.38518700	-1.98610300	3.22887100
H	-14.47086600	0.03416000	4.34821400
H	-13.68090100	1.52328500	3.79301900
H	-12.72086300	0.22678700	4.51895500
H	-14.59612600	-0.34620600	0.65027600
H	-15.52529400	-0.33178600	2.15215500
H	-14.83249900	1.17326800	1.53959800
H	-5.57401600	1.04185500	-3.81165900
H	-3.28036100	0.29116200	-3.41562100
H	0.01470600	-6.97513100	-3.50533700
H	-3.64996700	-5.21134700	-2.10457900
H	1.30000700	-5.49446400	-2.02624800
H	1.27319800	-3.59077600	-0.23396600
H	-3.51386000	-3.36596300	-0.36168000
H	-3.67055500	-3.81394000	2.20040600
H	-2.50103800	-4.27402100	4.31207000
H	1.27559200	-4.08625100	2.26211600
H	-0.08473300	-4.78766300	6.87966100
H	-1.20500900	-3.63938800	6.13590400
H	-1.41860200	-5.38493000	5.89020500
H	1.51467700	-6.11953400	5.52659300
H	1.66815100	-5.87434300	3.78004800
H	0.23433400	-6.67951400	4.43170600
H	0.81557700	-2.43680600	5.07310200
H	1.82694700	-3.60705900	5.94399200
H	2.03842700	-3.37878700	4.20659400
H	-2.81351700	-7.51110200	-6.01765500
H	-2.49985700	-5.81864000	-5.58362800
H	-1.20102100	-7.01463000	-5.48387000
H	-2.89065100	-9.30380900	-4.18780100
H	-2.59732000	-8.85301100	-2.49617300

H	-1.27018300	-8.82791100	-3.66482100
H	-4.51845400	-5.97538900	-4.02246300
H	-4.70188800	-7.68074700	-4.44187300
H	-4.55936200	-7.20818600	-2.74526400
H	1.67649400	0.10179900	-3.16896300
H	4.04956100	0.69799400	-3.25399100
H	10.25731200	-0.74629100	3.94579600
H	6.10544300	0.15584300	3.26987500
H	10.85215000	0.44483100	1.88151700
H	10.06001200	1.81776600	-0.18461100
H	5.48059000	1.49248900	1.19556700
H	5.23337300	4.09132800	1.23019400
H	6.28983800	6.28730300	0.90726200
H	9.97931400	4.36273700	-0.17811800
H	8.50618600	8.64498700	-1.10706400
H	8.13653400	7.10370500	-1.90439400
H	7.01782000	7.77449400	-0.71024900
H	9.18548900	8.68259100	1.35371400
H	9.25252200	7.17031500	2.28006200
H	7.69284900	7.83546800	1.77741600
H	10.91633000	6.31896200	0.51695000
H	10.45615500	6.31058300	-1.19860100
H	10.71616600	7.84081300	-0.35768800
H	8.56107800	-2.32248700	6.65806300
H	9.39878900	-2.53451200	5.11528100
H	9.65860500	-1.06625500	6.07988600
H	6.77367300	-0.62953700	6.95903700
H	6.20443600	0.35754100	5.60480000
H	7.79983300	0.66232800	6.30457400
H	7.11035700	-2.93651300	3.99541600
H	6.40193200	-2.77118800	5.61551600
H	5.78452400	-1.79994400	4.27626000
H	8.74707800	1.09157400	-4.73510800
H	10.79833100	0.94311200	-6.07719700
H	12.95345800	0.21500900	-5.06989300

PTA-Ph

C	-10.16485800	1.36417600	-0.81979500
C	-9.90482600	0.07347000	-0.49918700
C	-8.51867700	-0.17612000	-0.24685800
C	-7.72992900	0.92568600	-0.38783700
S	-8.70958100	2.30530400	-0.83194600
H	-11.11330900	1.82632700	-1.05438900
C	-6.27345600	1.04075600	-0.22777800
C	-8.75757600	-4.04205600	-2.79036800
C	-10.10800400	-3.81581200	-3.07315300
C	-10.79391100	-2.88175000	-2.28491500
C	-10.14567500	-2.20824800	-1.26240500
C	-8.79395700	-2.44937400	-0.99550600
C	-8.09985500	-3.36954400	-1.76198600
C	-8.23979600	-1.61427200	0.15341900
C	-10.77056400	-1.16294200	-0.33948200
C	-9.15012200	-1.88566300	1.34678600
C	-10.50579100	-1.64676800	1.08293700
C	-11.43836500	-1.85196700	2.07994700
C	-11.02142800	-2.29770900	3.33894600
C	-9.67858600	-2.54275800	3.61844400
C	-8.74447800	-2.32580500	2.59038100
C	-9.18609100	-3.04033400	4.98341000
C	-10.78177100	-4.57832700	-4.22230000
C	-10.32982700	-3.19951200	5.99412400
C	-8.50645000	-4.41134800	4.80547800
C	-8.17234900	-2.03382300	5.56017300
C	-10.06291200	-4.24386700	-5.54314400
C	-10.69029900	-6.09272700	-3.95582900
C	-12.26372000	-4.21000700	-4.37621800
C	-5.69379300	2.13303900	0.42740300
C	-4.31900300	2.21292000	0.59473500
C	-4.05547500	0.11484700	-0.55035200
C	-5.42973900	0.03920200	-0.72467100
C	-2.02333600	1.31101000	0.29441500
C	-1.21990400	2.39274200	0.09975600

C	0.15090300	2.15236500	0.41642400
C	0.40302700	0.88717000	0.84986400
S	-1.07698600	-0.03910600	0.87974400
C	-3.47740100	1.20426400	0.10980500
C	1.70534400	0.32581700	1.23512000
C	0.34737500	6.29364800	2.49820300
C	-0.99553800	6.54732200	2.20245300
C	-1.61414200	5.74449800	1.23451500
C	-0.90974700	4.73556500	0.59785400
C	0.43308400	4.49733700	0.90842900
C	1.06138400	5.28009600	1.86136900
C	1.04776900	3.34207800	0.12703500
C	-1.46432500	3.78434000	-0.45881800
C	0.81567700	3.65313000	-1.34721600
C	-0.52917600	3.89304300	-1.66031100
C	-0.88086500	4.17987300	-2.96421400
C	0.10596900	4.22632200	-3.95476100
C	1.44722000	3.98414200	-3.66258200
C	1.78476800	3.69408100	-2.32910500
C	2.55141400	4.00636700	-4.72720600
C	-1.73520900	7.67687200	2.93231900
C	2.01709700	4.39593900	-6.11195900
C	3.63459200	5.02614700	-4.32765600
C	3.18029600	2.60388900	-4.83018900
C	-1.73121400	7.39445600	4.44658500
C	-1.02373200	9.01483700	2.65590600
C	-3.19477100	7.80665300	2.47506100
C	2.16273500	-0.89454900	0.72819900
C	3.44343000	-1.34326200	1.01840600
C	4.31026400	-0.57678900	1.80695900
C	3.83355700	0.61709100	2.35792400
C	2.54682300	1.05630900	2.08323600
C	5.70870700	-0.99574300	1.97709200
C	6.57607200	-1.38369600	1.00250200
C	7.89078600	-1.66541200	1.48053200
C	8.02871500	-1.51968800	2.82704900

S	6.51312700	-1.00010100	3.52805400
C	9.21231000	-1.80058800	3.65410300
C	9.59077100	1.02556800	-1.90758700
C	8.30387700	1.30608000	-2.37863400
C	7.25610900	0.48808700	-1.93764900
C	7.49982500	-0.55895900	-1.06446300
C	8.79079900	-0.82131300	-0.59903800
C	9.83991900	-0.02402000	-1.02432900
C	8.86667200	-2.00175700	0.36659300
C	6.44904400	-1.51023900	-0.50513300
C	8.23247700	-3.18432800	-0.35750000
C	6.93704700	-2.91931200	-0.82626600
C	6.24413900	-3.90780100	-1.49585900
C	6.83745600	-5.15934700	-1.69563800
C	8.11971600	-5.44300900	-1.23021800
C	8.80991500	-4.42285100	-0.55141000
C	8.78922100	-6.81094800	-1.41358700
C	8.08320700	2.46772800	-3.35740800
C	7.92870900	-7.77206300	-2.24459800
C	10.14030000	-6.63427100	-2.13210300
C	9.02297600	-7.44380400	-0.02834300
C	8.60732500	3.77666100	-2.73752900
C	8.84481100	2.17606100	-4.66407700
C	6.60006900	2.66430600	-3.69939700
C	9.94388800	-2.97348400	3.42689700
C	11.08114100	-3.25361000	4.17482600
C	11.49557000	-2.37551800	5.17243800
C	10.76530200	-1.21578000	5.41719700
C	9.63350300	-0.92737400	4.66262700
H	12.38123400	-2.59632100	5.75953200
H	-8.19617700	-4.75795500	-3.38334600
H	-11.84273800	-2.67208400	-2.46510800
H	-7.04975600	-3.56784800	-1.56462700
H	-7.19018100	-1.81797700	0.36870200
H	-11.82887600	-0.98975000	-0.54293500
H	-12.49285000	-1.66956200	1.89231700

H	-11.77355600	-2.45048500	4.10416100
H	-7.68683100	-2.50317200	2.76652700
H	-9.92822400	-3.55378900	6.94857600
H	-10.83974100	-2.24888100	6.17991300
H	-11.07109300	-3.93044900	5.65582100
H	-8.13644700	-4.78194800	5.76769300
H	-7.65646500	-4.35593300	4.11946800
H	-9.21335500	-5.14433500	4.40446900
H	-7.30434700	-1.91019100	4.90666300
H	-8.63472100	-1.05095000	5.69312600
H	-7.80862900	-2.37749600	6.53461600
H	-10.53247100	-4.77492900	-6.37830500
H	-10.11164500	-3.17016800	-5.74878100
H	-9.00820500	-4.53179600	-5.51381200
H	-11.16807200	-6.65072700	-4.76841800
H	-11.19271000	-6.35232400	-3.01897800
H	-9.65267600	-6.43063500	-3.88394400
H	-12.39769800	-3.14648500	-4.59818400
H	-12.69848400	-4.77710400	-5.20498600
H	-12.83592200	-4.44986700	-3.47419500
H	-6.33182000	2.91069600	0.83633500
H	-3.88616400	3.04856400	1.13656600
H	-3.41611500	-0.66343400	-0.95598200
H	-5.85861800	-0.78954400	-1.27945300
H	0.85771700	6.89801900	3.24227700
H	-2.65407600	5.90215700	0.96882800
H	2.10532000	5.10871400	2.10980300
H	2.09864800	3.16979700	0.36330400
H	-2.50719600	3.98035400	-0.71197900
H	-1.91933700	4.36563600	-3.22351300
H	-0.19798200	4.45297600	-4.96996800
H	2.81857400	3.49430000	-2.05881400
H	2.83989000	4.40946500	-6.83338600
H	1.27289700	3.67997600	-6.47475700
H	1.56391200	5.39225600	-6.10483200
H	4.41981200	5.06165600	-5.09067900

H	4.10945400	4.76768600	-3.37701600
H	3.20631100	6.02829500	-4.22845200
H	3.61393700	2.28527000	-3.87784200
H	2.42997000	1.86306800	-5.12280700
H	3.97989100	2.59910800	-5.57907200
H	-2.25563600	8.19245300	4.98341300
H	-2.23232900	6.44652300	4.66525600
H	-0.71488200	7.33506200	4.84618300
H	-1.54499100	9.83535300	3.16098000
H	-1.00421500	9.22903400	1.58293300
H	0.00942900	9.00536600	3.01462900
H	-3.76433300	6.89362500	2.67639700
H	-3.67825100	8.62443000	3.01820200
H	-3.26813000	8.03127600	1.40619400
H	1.51754300	-1.48163200	0.08166100
H	3.78951800	-2.29305100	0.62214900
H	4.48759500	1.21654100	2.98382500
H	2.18291100	1.98183300	2.51905800
H	10.42724400	1.63719100	-2.23259600
H	6.23824800	0.66173600	-2.26979700
H	10.85023000	-0.21083100	-0.67144600
H	9.88100800	-2.20079000	0.71494100
H	5.44690300	-1.30461500	-0.88336800
H	5.24172700	-3.71866800	-1.87054200
H	6.27132100	-5.91535200	-2.22705100
H	9.81250700	-4.60047900	-0.17033400
H	8.44996200	-8.72739200	-2.35953100
H	6.96806000	-7.97732700	-1.76200400
H	7.73555300	-7.37537600	-3.24638700
H	10.62206900	-7.60710000	-2.27739900
H	10.82806800	-6.00650900	-1.55870700
H	10.00093000	-6.17004800	-3.11316600
H	9.66461000	-6.81676900	0.59788800
H	8.07390700	-7.58374800	0.49806900
H	9.50625600	-8.42137200	-0.13205600
H	8.44078800	4.61361500	-3.42421800

H	8.09140400	3.99533600	-1.79759600
H	9.67950000	3.72709900	-2.52830100
H	8.70458600	2.99335600	-5.38005900
H	8.48270600	1.25144800	-5.12400100
H	9.91840800	2.06404000	-4.48749200
H	6.00429600	2.88629900	-2.80734100
H	6.48611800	3.50660100	-4.38891200
H	6.17287600	1.78282200	-4.18743700
H	9.59769300	-3.67517400	2.67356800
H	11.63565300	-4.16792500	3.98834100
H	11.08288900	-0.52643800	6.19302400
H	9.07948200	-0.01065900	4.84137500

PTA-Th

C	-9.16416600	0.77391600	1.17851100
C	-8.88839600	-0.48164000	0.74861000
C	-7.48736900	-0.73545900	0.62137900
C	-6.70600900	0.32156800	0.97556500
S	-7.70781500	1.67211800	1.46147100
C	-5.25095800	0.36466300	1.03183600
C	-8.28466000	-2.58360000	-3.56299000
C	-9.65971500	-2.35714900	-3.44583300
C	-10.16764000	-2.06375400	-2.17342300
C	-9.32518600	-2.00220900	-1.07479200
C	-7.95364500	-2.23369200	-1.21294300
C	-7.43299300	-2.52470400	-2.46200500
C	-7.17481000	-2.12091800	0.09334900
C	-9.74101500	-1.67559200	0.35815100
C	-7.85477200	-3.07117200	1.07312000
C	-9.22896000	-2.82930400	1.21793500
C	-9.96655100	-3.61226100	2.08315000
C	-9.33801700	-4.63496700	2.80209000
C	-7.97463800	-4.88988800	2.66991600
C	-7.23992100	-4.08161500	1.78431600
C	-7.25747400	-6.01008000	3.43411200
C	-10.54837200	-2.42625700	-4.69545300
C	-8.19842400	-6.76062300	4.38587500

C	-6.68288500	-7.02206200	2.42460000
C	-6.11045100	-5.40992100	4.26926200
C	-10.07844600	-1.36711900	-5.71069700
C	-10.43657400	-3.82758100	-5.32533100
C	-12.02594000	-2.16151100	-4.37593700
S	-4.33166400	1.66060600	0.32895800
C	-2.82499000	0.95103400	0.83254900
C	-3.04489800	-0.24089600	1.47382000
C	-4.42049600	-0.57066200	1.59039300
C	-1.54737100	1.58052100	0.53301100
C	-1.20362900	2.89915800	0.46176300
C	0.17275400	3.11315700	0.14939800
C	0.88075900	1.96442800	-0.03631900
S	-0.15942700	0.57597200	0.17596600
C	2.27567700	1.82978200	-0.42765500
C	0.40041500	6.26487100	3.56681900
C	-0.95015300	6.07832700	3.87946500
C	-1.74906700	5.40138100	2.94920900
C	-1.20791700	4.94155900	1.75945700
C	0.14194000	5.14242400	1.46287500
C	0.94947600	5.80410900	2.37203100
C	0.56180400	4.57847600	0.11039400
C	-1.96613300	4.19775500	0.66486300
C	-0.38177800	5.19459900	-0.91857700
C	-1.73658900	4.98441400	-0.62278900
C	-2.70217000	5.46573000	-1.48349800
C	-2.31948300	6.15932700	-2.63672700
C	-0.97881500	6.38064600	-2.94577200
C	-0.01031800	5.88078800	-2.05705600
C	-0.52703300	7.13629500	-4.20178900
C	-1.50215900	6.60948800	5.20935700
C	-1.71188100	7.59795400	-5.06093800
C	0.28026000	8.38061100	-3.78549600
C	0.35867700	6.21411400	-5.06117000
C	-0.76927500	5.91969900	6.37558900
C	-1.27509900	8.13117500	5.28577700

C	-3.00579300	6.34277300	5.36329500
S	3.33641900	0.66968700	0.31266900
C	4.66718500	1.14273800	-0.70239000
C	4.29984100	2.17067900	-1.53140900
C	2.94180200	2.55500500	-1.38123900
C	5.96735200	0.49596800	-0.59617400
C	6.28041400	-0.82024700	-0.42299400
C	7.68468400	-1.07370200	-0.40721400
C	8.44881900	0.04569900	-0.54619700
S	7.42649600	1.45480600	-0.70213200
C	9.90149600	0.14024100	-0.51632800
C	7.02546100	-4.56931800	-3.40593300
C	5.65152500	-4.33618100	-3.42787900
C	5.10794600	-3.52510900	-2.41624900
C	5.91412700	-2.98191700	-1.43651400
C	7.29360200	-3.22720400	-1.43052100
C	7.84701400	-4.02021400	-2.41590400
C	8.02841900	-2.54523000	-0.28147200
C	5.45190800	-2.08651500	-0.29162600
C	7.32855700	-2.99708900	0.99641000
C	5.94817700	-2.74807000	0.99077400
C	5.19151100	-3.09405300	2.09223200
C	5.80834800	-3.68865100	3.19828400
C	7.17824500	-3.94259400	3.22260900
C	7.93152000	-3.58205700	2.09135500
C	7.88671000	-4.58523500	4.42164200
C	4.72204700	-4.92042200	-4.49932700
C	6.91410900	-4.92673100	5.55841800
C	8.57688400	-5.88707200	3.97255300
C	8.94248500	-3.60620900	4.97010900
C	5.48024900	-5.76698200	-5.53039400
C	3.66596200	-5.81625000	-3.82446000
C	4.01850200	-3.77063000	-5.24537200
S	10.79629800	1.11111200	-1.65160300
C	12.31749700	0.64421600	-0.95727600
C	12.12549300	-0.22040400	0.08084900

C	10.75372600	-0.50365100	0.33913000
C	13.61063300	1.18098300	-1.48797600
H	-10.12644600	1.23595100	1.34816900
H	-7.85683900	-2.80883700	-4.53533200
H	-11.22676500	-1.87835800	-2.03031200
H	-6.36843700	-2.70121000	-2.58605700
H	-6.10722200	-2.31150100	-0.02638400
H	-10.81377200	-1.50570700	0.46596400
H	-11.03145100	-3.43840800	2.20997800
H	-9.94185700	-5.23486500	3.47277300
H	-6.17468800	-4.24882800	1.64602000
H	-7.64148800	-7.54118700	4.91337000
H	-8.62715500	-6.09189400	5.13915100
H	-9.01837100	-7.24667800	3.84775900
H	-6.16756800	-7.83402800	2.94920600
H	-5.96448100	-6.55332100	1.74592600
H	-7.48166000	-7.45853400	1.81723000
H	-5.37058000	-4.90548800	3.64121900
H	-6.49317700	-4.68004300	4.98923900
H	-5.59172800	-6.19949500	4.82363700
H	-10.70065600	-1.40029000	-6.61176900
H	-10.14822700	-0.36278800	-5.28179000
H	-9.04077400	-1.53044500	-6.01462000
H	-11.06472800	-3.89338900	-6.22037500
H	-10.76280000	-4.59709000	-4.61901400
H	-9.40925900	-4.05768000	-5.62116100
H	-12.17740900	-1.16312700	-3.95326400
H	-12.61604600	-2.22427500	-5.29541000
H	-12.42676900	-2.89945700	-3.67349900
H	-2.24069400	-0.84339800	1.87921200
H	-4.79959800	-1.44743800	2.10211800
H	1.04767000	6.78333800	4.26796900
H	-2.80172000	5.22778900	3.14397200
H	2.00244200	5.96394100	2.15834600
H	1.61665500	4.74403300	-0.11256600
H	-3.02212500	4.06299300	0.89982000

H	-3.75559200	5.30421000	-1.27253900
H	-3.09762500	6.52425000	-3.29667400
H	1.04704900	6.03292100	-2.25927500
H	-1.34037700	8.12765800	-5.94350900
H	-2.31238200	6.75230200	-5.41090500
H	-2.36538800	8.28383000	-4.51283000
H	0.60911700	8.93393500	-4.67178000
H	1.17139700	8.11262700	-3.21031100
H	-0.32859300	9.04881100	-3.16885700
H	1.25498600	5.89420900	-4.52185600
H	-0.19060400	5.31627600	-5.36064300
H	0.68438400	6.73594500	-5.96742700
H	-1.14910300	6.28904400	7.33446200
H	-0.91984000	4.83638900	6.34043100
H	0.30735500	6.10980100	6.34558000
H	-1.67332600	8.52889900	6.22553000
H	-1.77788800	8.63994600	4.45746100
H	-0.21248100	8.38577000	5.23935500
H	-3.23096700	5.27155000	5.35606400
H	-3.35461300	6.74703500	6.31858100
H	-3.58555200	6.82407000	4.56915400
H	4.97865700	2.60680600	-2.25449000
H	2.44927600	3.30724400	-1.98600200
H	7.48535100	-5.18787000	-4.16772900
H	4.04162000	-3.31609600	-2.39572300
H	8.91552200	-4.21610100	-2.42741800
H	9.10114800	-2.74299100	-0.28215100
H	4.37486900	-1.91619300	-0.29672300
H	4.12180600	-2.90433400	2.10481100
H	5.19125400	-3.95035100	4.04983800
H	9.00341200	-3.76246300	2.06777600
H	7.46382300	-5.38333500	6.38738800
H	6.41413500	-4.03363700	5.94621200
H	6.14940800	-5.64033500	5.23585700
H	9.08432200	-6.35890700	4.82097100
H	9.32546100	-5.70434700	3.19632200

H	7.84526000	-6.59629500	3.57345500
H	9.69765900	-3.36164000	4.21754300
H	8.47401600	-2.67099700	5.29145200
H	9.45781000	-4.04602200	5.83082500
H	4.77654200	-6.15654700	-6.27255500
H	6.23228100	-5.17759300	-6.06448800
H	5.97769100	-6.62368500	-5.06463400
H	2.98820700	-6.23688200	-4.57530900
H	3.06008000	-5.25722700	-3.10577900
H	4.14262600	-6.64393600	-3.29025100
H	4.75046200	-3.11687400	-5.72939400
H	3.35118300	-4.16975900	-6.01695500
H	3.41558300	-3.15603300	-4.57098000
H	12.94627000	-0.63318000	0.65617700
H	10.39919100	-1.13508500	1.14545700
H	14.44551500	0.71450100	-0.95963700
H	13.73034700	0.97423600	-2.55583200
H	13.68624500	2.26394200	-1.34786500

Supplementary References

1. Ali, S. *et al.* Gas Phase Photocatalytic CO2 Reduction, “A Brief Overview for Benchmarking”. *Catalysts* **9**, 727 (2019).
2. Urbani, M. *et al.* Heteroleptic Ru(ii)-bipyridine complexes based on hexylthioether-, hexyloxy- and hexyl-substituted thienylenevinylenes and their application in dye-sensitized solar cells. *Phys. Chem. Chem. Phys.* **18**, 11901–11908 (2016).
3. Amou, S. *et al.* Head-to-Tail Regioregularity of Poly(3-hexylthiophene) in Oxidative Coupling Polymerization with FeCl 3. *J Polym Sci A Polym Chem* **37**, 1943–1948 (1999).
4. Sahu, H., Gupta, S., Gaur, P. & Panda, A. N. Structure and optoelectronic properties of helical pyridine–furan, pyridine–pyrrole and pyridine–thiophene oligomers. *Phys. Chem. Chem. Phys.* **17**, 20647–20657 (2015).
5. Yano, T., Ebizuka, M., Shibata, S. & Yamane, M. Anomalous chemical shifts of Cu 2p and Cu LMM Auger spectra of silicate glasses. *J. Electron Spectros. Relat. Phenomena* **131**–**132**, 133–144 (2003).

6. Biesinger, M. C. Advanced analysis of copper X-ray photoelectron spectra. *Surf. Interface Anal.* **49**, 1325–1334 (2017).
